# Modified STRONGkids outperforms PYMS for WHO-defined malnutrition: a screening-to-action pathway for pediatric wards

**DOI:** 10.3389/fped.2025.1628856

**Published:** 2026-02-12

**Authors:** Hong Chen, Yu Zhang, Hongwei Han

**Affiliations:** Department of Pediatrics, Wenzhou People’s Hospital, Wenzhou, Zhejiang, China

**Keywords:** digestive system diseases, nutritional, nutritional risk screening, PYMS, STRONGkids

## Abstract

**Objective:**

The objective of this paper was to understand the nutritional status of hospitalized children and explore the clinical application value of the modified STRONGkids and PYMS screening tools.

**Methods:**

Nutritional risk screening was performed using the modified STRONGkids and PYMS tools on 302 children admitted to the pediatric department of our hospital between April 2021 and March 2023. Differences in clinical outcomes and biochemical indicators under different nutritional statuses were analyzed.

**Results:**

Overall, 302 children were included in the current study. The modified STRONGkids screening tool identified 18 (5.96%) children at high risk of malnutrition, 210 (69.54%) at moderate risk, and 74 (24.50%) at low risk. The PYMS screening tool identified 16 (5.30%) children at high risk, 152 (50.33%) at moderate risk, and 134 (44.37%) at low risk. According to WHO nutritional assessment standards, 150 (49.67%) children were identified as borderline malnourished or malnourished. Both the modified STRONGkids and PYMS evaluations indicated that children with digestive system diseases had a significantly higher risk of malnutrition compared with those who had other systemic diseases. Moreover, children under 1 year of age had a significantly higher risk of malnutrition compared with other age groups (*P* < 0.05). High-risk children identified by both screening methods had higher rates of hospital-acquired infections, longer hospital stays, and lower levels of hemoglobin and prealbumin compared with those at moderate and low risk (*P* < 0.05). The modified STRONGkids tool demonstrated higher sensitivity and accuracy in predicting malnutrition in hospitalized children compared than the PYMS tool (*P* < 0.05).

**Conclusion:**

Children with digestive system diseases and those under 1 year of age are at high risk for malnutrition. Both the modified STRONGkids and PYMS tools can effectively identify children at risk of malnutrition, with the modified STRONGkids tool demonstrating better application results.

## 1 Introduction

Malnutrition is a significant public health issue with a high incidence among hospitalized children. It not only impairs their growth and development but also increases their risk of infections and mortality, prolonging hospital stays and adversely impacting their prognosis. Therefore, early screening and assessment of malnutrition risk in hospitalized children can improve clinical outcomes (1–3). Currently, six main tools are available for assessing malnutrition risk in hospitalized children, among which STRONGkids and PYMS are widely used. These tools facilitate the early detection and treatment of malnutrition in hospitalized children. However, existing studies on STRONGkids and PYMS have primarily focused on single-disease categories, lacking comprehensive research across multiple diseases (4, 5). This study aimed to explore the clinical application value of the modified STRONGkids and PYMS tools in assessing malnutrition risk among hospitalized children and to evaluate the awareness of malnutrition risk among medical staff. The findings are reported as follows.

## 2 Subjects and methods

### 2.1 Selection criteria for cases

Using a point-continuous sampling method, we collected data from 302 newly admitted children hospitalized in five different pediatric departments of our hospital between April 2021 and March 2023. All disease diagnoses were based on “Pediatrics” (6). Types of diseases included the following: digestive system: hepatitis, gastrointestinal bleeding, diarrheal diseases, and peptic ulcers; respiratory system: bronchiolitis, bronchial asthma, pneumonia, and bronchial foreign bodies; infectious diseases: hand-foot-and-mouth disease, infectious mononucleosis, and sepsis; neurological disorders: acute bacterial meningitis, viral encephalitis, and Guillain–Barré syndrome; pediatric surgery: inguinal hernia, intussusception, congenital anorectal malformations, appendicitis, and congenital hypertrophic pyloric stenosis; and vasculitis/immunologic: Kawasaki disease.

Inclusion Criteria: hospital stay >24 h, corrected gestational age >1 month for preterm infants, age between 29 days and 14 years, and informed consent signed by the guardians.

Exclusion criteria: received parenteral nutrition support within the past 3 months, hospital stay <24 h, received enteral nutrition support within the past 3 months, and refusal to participate in the study. This study was reviewed and approved by the Wenzhou People's Hospital Ethics Committee [Lun Shen (2020) No. (192)]. All procedures were conducted in accordance with the ethical standards outlined in the Declaration of Helsinki, and written informed consent was obtained from all participants prior to their participation.

### 2.2 Research tools

All subjects underwent relevant tests and were screened for malnutrition risk using the modified STRONGkids and PYMS tools. Modified STRONGkids scoring (7) includes four dimensions: weight loss/growth retardation, subjective clinical assessment, nutritional intake and loss, and potential disease risk. Dimensions 1 and 3 were assessed by a professional research team in consultation with parents, while dimensions 2 and 4 were evaluated by pediatricians. Scores range from 0 to 5, corresponding to low (0 points), moderate (1–3 points), and high (4–5 points) nutritional risk. PYMS scoring (8) includes four dimensions: changes in food intake over the past week, BMI below the median by 2 SD, recent weight loss, and impact of disease on nutritional status for at least 1 week. Each dimension scores 0–2 points, corresponding to low (0 points), moderate (1 point), and high (≥2 points) nutritional risk.

### 2.3 Data collection

Clinical data and medical history of all subjects were collected according to nutritional assessment standards. Nutritional risk was determined based on the scoring results and the WHO nutritional assessment standards.

### 2.4 Malnutrition assessment

Malnutrition was assessed according to the WHO (9) standards, using height-for-age Z-score, weight-for-age Z-score, BMI-for-age Z-score, and weight-for-height Z-score. A Z-score <−2 in any of these parameters confirmed malnutrition.

### 2.5 Statistical analysis

Data analysis was performed using SPSS 19.0. Measurement data were expressed as mean ± standard deviation (SD), and comparisons between groups were made using the *t*-test. Categorical data were expressed as percentages, and comparisons between groups were made using the chi-square test. Diagnostic efficacy was analyzed using receiver operating characteristic (ROC) curves to obtain the area under the curve (AUC), confidence intervals, sensitivity, specificity, and accuracy. A *P*-value < 0.05 was considered statistically significant.

## 3 Results

### 3.1 Study population

From April 2021 to March 2023, a total of 302 hospitalized children were enrolled from five pediatric departments. The cohort included 168 boys (55.6%) and 134 girls (44.4%), aged 29 days to 14 years (mean ± SD: 5.2 ± 3.6 years).

By age group, 89 (29.5%) were <1 year, 96 (31.8%) were 1–3 years, and 117 (38.7%) were >3 years.

The most common admission diagnoses were respiratory system diseases (*n* = 172, 57.0%), followed by digestive system diseases (*n* = 42, 13.9%), infectious diseases (*n* = 37, 12.3%), neurological disorders (*n* = 25, 8.3%), pediatric surgical diseases (*n* = 20, 6.6%), and vasculitis/immunologic diseases (*n* = 6, 2.0%).

### 3.2 Evaluation of nutritional risk and incidence among hospitalized children using two screening tools

The modified STRONGkids screening tool identified 18 children (5.96%) at high risk of malnutrition, 210 children (69.54%) at moderate risk, and 74 children (24.50%) at low risk. The PYMS screening tool identified 16 children (5.30%) at high risk of malnutrition, 152 children (50.33%) at moderate risk, and 134 children (44.37%) at low risk. According to WHO nutritional assessment standards, 150 children (49.67%) were identified as borderline malnourished or malnourished.

### 3.3 Distribution differences in nutritional risk among different systemic diseases

As shown in Table 1, both the modified STRONGkids and PYMS evaluations indicated that children with digestive system diseases had a significantly higher risk of malnutrition compared with those with respiratory system diseases, neurological disorders, infectious diseases, and surgical conditions, with statistical significance (*P* < 0.05).

**Table 1:** Distribution differences of nutritional risks for different systemic diseases (*n*, %).

Systemic diseases	Number of cases	STRONGkids		PYMS	
		Low to medium risk	High risk	Low to medium risk	High risk
Respiratory system diseases	172	169 (98.26)	3 (1.74)	170 (98.84)	2 (1.16)
Digestive system diseases	42	32 (76.19)	10 (23.81)	34 (80.95)	8 (19.05)
Neurological disorders	25	23 (92.00)	2 (8.00)	23 (92.00)	2 (8.00)
Infectious diseases	37	35 (94.59)	2 (5.41)	34 (91.89)	3 (8.11)
Vasculitis/Immunologic	6	5 (83.33)	1 (16.66)	4 (66.66)	2 (33.33)
*x*^2^ value		28.805		24.240	
*P-*value		0.001		0.001	

### 3.4 Distribution differences of nutritional risks among different age groups

As shown in Table 2, the improved version of STRONGkids and PYMS evaluation showed that the risk of malnutrition in children under 1 year of age was significantly higher than that in children aged 1–3 years and over 3 years of age, with statistical differences (*P* < 0.05).

**Table 2:** Distribution differences of nutritional risks among different age groups (*n*, %).

Age	*N*	STRONGkids		PYMS	
		Low to medium risk	High risk	Low to medium risk	High risk
<1 year	89	76 (85.39)	13 (14.61)	80 (89.89)	9 (10.11)
1–3 years	96	93 (96.88)	3 (3.13)	94 (97.92)	2 (2.08)
>3 years	117	115 (98.29)	2 (1.71)	112 (95.73)	5 (4.27)
*x*^2^ value		7.706		5.324	
*P-*value		0.006		0.021	

### 3.5 Comparison of clinical outcomes among children with different nutritional risk levels

As shown in Table 3, among the 302 hospitalized children, 31 experienced varying degrees of hospital-acquired infections. The modified STRONGkids and PYMS screening results indicated that the hospital-acquired infection rates for children at high nutritional risk were 66.67% and 62.50%, respectively. In contrast, the infection rates for children at moderate to low nutritional risk were 6.69% and 7.34%, respectively. Both screening methods suggested that children at high nutritional risk had significantly higher rates of hospital-acquired infections. In addition, children identified as high nutritional risk by both the modified STRONGkids and PYMS tools had longer hospital stays compared with those at moderate to low risk, with statistical significance (*P* < 0.05).

**Table 3:** Comparison of clinical outcomes in children with different levels of nutritional risk.

Screening tools	Group	*N*	Nosocomial infection	*x* ^2^	*P*	Hospitalization time (days)	*t*	*P*
STRONGkids	Low to medium risk	284	19 (6.69)	66.104	0.001	3.12 ± 0.58	14.828	0.001
	High risk	18	12 (66.67)			5.23 ± 0.67		
PYMS	Low to medium risk	286	21 (7.34)	50.046	0.001	3.18 ± 0.60	14.375	0.001
	High risk	16	10 (62.50)			5.36 ± 0.72		

### 3.6 Comparison of biochemical indicators in children with different nutritional risk levels

As shown in Table 4, the hemoglobin and prealbumin levels of children at high risk of malnutrition screened by the improved STRONGkids and PYMS tools were lower than those of children at medium to low risk, with statistical differences (*P* < 0.05).

**Table 4:** Comparison of biochemical indicators in children with different nutritional risk levels ($\bar{x}\pm s$).

Screening tools	Group	*N*	Hemoglobin (g/L)	*t*	*P*	Prealbumin (mg/L)	*t*	*P*
STRONGkids	Low to medium risk	284	102.38 ± 14.25	3.036	0.001	96.01 ± 9.12	7.299	0.001
	High risk	18	92.01 ± 10.27			80.01 ± 7.12		
PYMS	Low to medium risk	286	103.98 ± 15.01	3.359	0.001	95.58 ± 9.36	5.942	0.001
	High risk	16	91.20 ± 10.31			81.37 ± 8.27		

### 3.7 ROC curve analysis of the predictive value of two tools for malnutrition in hospitalized children

**Figure 1:**
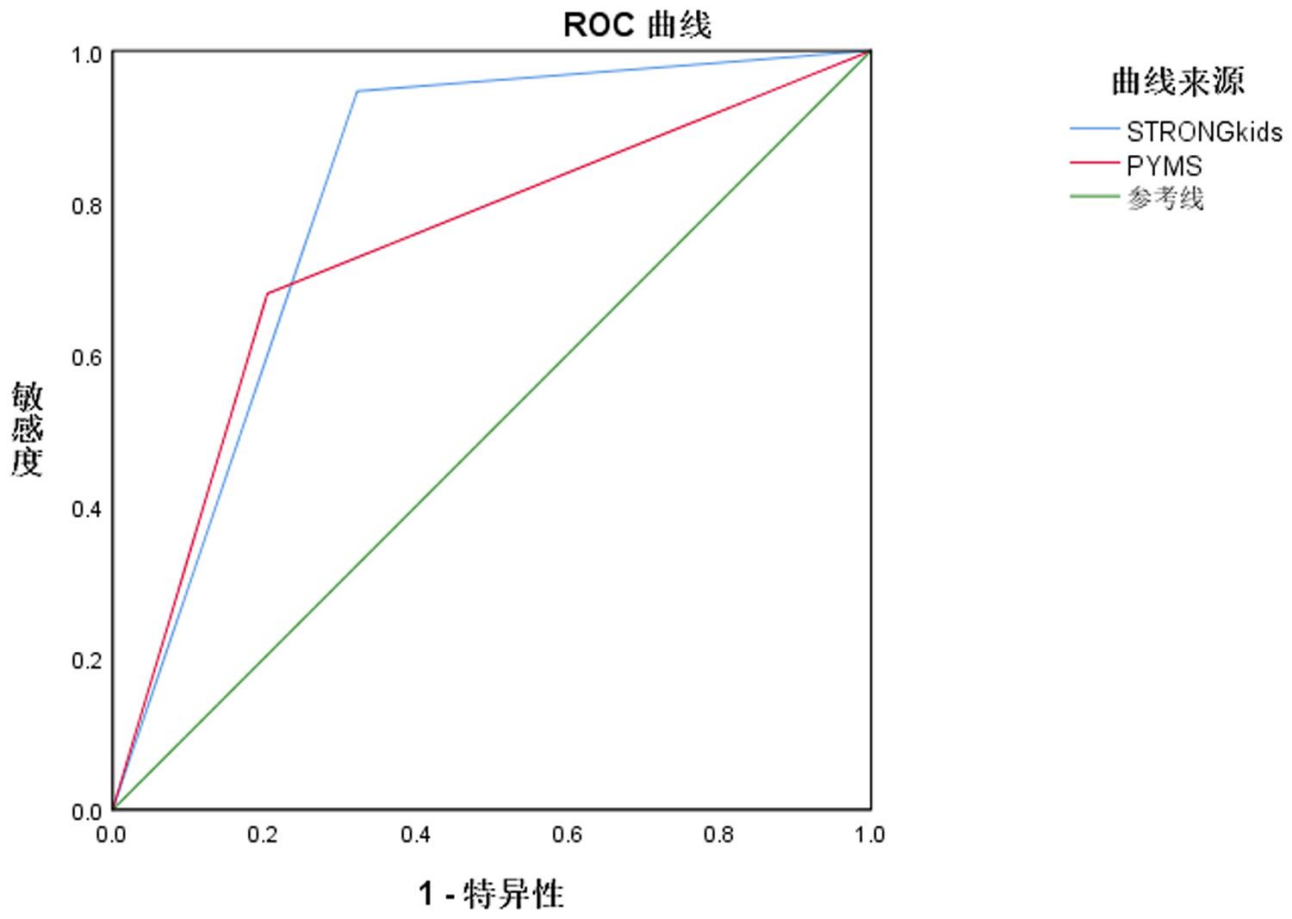
ROC curves of two tools for predicting malnutrition in hospitalized children.

As shown in Table 5, compared with PYMS screening, improved STRONGkids screening demonstrated higher sensitivity and accuracy in predicting malnutrition in hospitalized children, with statistical differences (*P* < 0.05) (Figure 1).

**Table 5:** ROC curve analysis of the predictive value of two tools for malnutrition in hospitalized children.

Index	Area under the curve (95% CI)	*P*	Sensitivity (%)	Specificity (%)	Accuracy (%)
STRONGkids	0.812 (0.761–0.863)	0.026	94.67 (142/150)	67.76 (103/152)	81.13 (245/302)
PYMS	0.738 (0.681–0.795)	0.029	68.00 (102/150)	79.61 (121/152)	73.84 (223/302)

Renal subgroup clinical data: Among 12 admissions, six cases were nephrotic, four cases were CKD, and two cases were AKI. The median age was 5.3 years, with 55% male. Serum albumin was 27 g/L. Dietary restrictions included protein restriction in 33% and sodium restriction in 50%. In this group, 25% were classified as high-risk by STRONGkids; 25% by PYMS; and 33% were identified as malnourished according to WHO criteria. Mean length of stay was 4.4 days, and hospital-acquired infection occurred in 8% of cases.

## 4 Discussion

We evaluated two pediatric nutrition screening tools against WHO-defined malnutrition and downstream clinical outcomes. Both the modified STRONGkids and PYMS tools identified nutritional risk to varying degrees, but the modified STRONGkids demonstrated superior discrimination and provided more informative risk stratification for hospital-acquired infection and length of stay. Risk gradients were most pronounced among infants younger than one year and children with digestive diseases. These findings support routine use of the modified STRONGkids tool, with targeted escalation for high-risk strata.

Nutrition is fundamental for maintaining various physiological functions of the body, and children have higher nutritional demands. Therefore, the risk of malnutrition is relatively high among children. Hospitalized children are at an increased nutritional risk due to factors such as digestive and absorption disorders, increased nutritional consumption, and reduced intake. Critically ill children are at an even higher nutritional risk, which not only affects their growth and development but also impacts their physiological development, cognitive development, and immune function, ultimately leading to adverse outcomes such as increased mortality and prolonged hospital stays (10–12). Therefore, it is crucial to clearly identify whether hospitalized children are at nutritional risk and provide timely and appropriate nutritional therapy to improve their health status and reduce the risk of adverse outcomes. Currently, nutritional risk screening methods for adult patients are relatively well developed, whereas pediatric nutritional screening methods remain fewer and outdated. Commonly used tools for screening nutritional risk in hospitalized children include PNRS, STAMP, PYMS, SGNA, and STRONGkids. Each of these tools has its own advantages and disadvantages, but there is still no universally accepted method for assessing the nutritional status of children. Thus, further research is needed to validate the effectiveness and feasibility of the existing screening methods (13, 14). Healthcare professionals play a crucial role in nutritional risk screening. If they lack awareness of the risk of malnutrition, the quality of their work may decline. Therefore, it is imperative to enhance clinical nutrition knowledge and awareness of nutritional risk screening of healthcare professionals. The absence of universally accepted screening tools and insufficient emphasis on the impact of malnutrition on clinical outcomes by healthcare professionals have resulted in limited research on nutritional risk screening for hospitalized children.

While adult nutritional risk screening tools have become more sophisticated in recent years, they are not suitable for pediatric populations. This is primarily because children's height and weight are constantly changing, and their etiological and pathological conditions differ from those of adults. Therefore, it is essential to find more appropriate nutritional risk screening tools for children.

The modified STRONGkids tool is time-efficient, practical, simple, highly correlated with clinical outcomes, and highly sensitive. However, its assessment requires professionals with specialized knowledge, demanding a higher level of expertise from clinicians or nurses. In addition, it requires family members to retrospectively calculate the child's weight changes, which may introduce calculation errors (15). The PYMS tool is a comprehensive screening tool that integrates nutritional issues with clinical assessments of children, offering high repeatability. However, it does not account for nutritional risks posed by potential diseases (16). Therefore, promoting the clinical application of these two nutritional risk screening tools should be tailored to actual circumstances to enhance clinical diagnostic accuracy and treatment levels and improve clinical outcomes for children.

In this study, both the modified STRONGkids and PYMS tools were used to assess the risk of malnutrition in children with different systemic diseases and ages. The results indicated that children with digestive system diseases and those under 1 year of age had a higher risk of malnutrition. The analysis suggested that digestive system diseases are more likely to cause symptoms such as indigestion, vomiting, loss of appetite, and diarrhea compared to other diseases, leading to reduced nutritional intake and increased risk of malnutrition. Younger children, who have not yet fully developed their immune systems, also grow rapidly and therefore have higher nutritional needs and weaker infection resistance, further increasing their risk of malnutrition (17, 18). In addition, children identified as high nutritional risk by the modified STRONGkids and PYMS tools had higher infection rates and longer hospital stays compared with those at moderate to low risk. Their hemoglobin and prealbumin levels were also lower than those of children at moderate to low risk. This is primarily because biochemical indicators can effectively reflect the nutritional status of children. Hemoglobin and prealbumin are commonly recognized indicators for assessing nutritional status, and their levels are inversely related to the risk of malnutrition. Infection rates and hospital stays can increase with the risk of malnutrition (19–21). Currently, there is a growing emphasis on nutritional risk in clinical practice, and most healthcare professionals recognize the importance of nutritional status for risk screening. The results of this study also revealed that among 80 healthcare professionals, eight were very knowledgeable about the risk of malnutrition and 52 were fairly knowledgeable about it.

## 5 Strengths and limitations

This study's strengths include a head-to-head evaluation of two pediatric nutrition screening tools within the same cohort, benchmarking against WHO anthropometric criteria, and linking screening strata to clinically relevant outcomes (hospital-acquired infection and length of stay), thereby enabling a pragmatic screening-to-action pathway. However, the observational, single-system design limits causal inference and generalizability, and both selection and spectrum biases are possible. Screening timing and re-screen cadence were not fully standardized, some inputs relied on parent report, and interrater reliability was not prospectively quantified, introducing potential misclassification. Outcome ascertainment relied on routine surveillance and may have underdetected infections; residual confounding by illness severity, treatment intensity, and staffing cannot be excluded despite adjustment. Several subgroup analyses were underpowered, no multiplicity adjustment was applied, and discrimination was estimated in sample without optimism correction or external validation, so performance may be overstated. The comparison was restricted to two tools, and implementation outcomes (time-to-complete, staff burden, workflow, costs) and the proposed decision thresholds were not prospectively tested. These constraints support cautious interpretation and motivate multicenter validation with predefined re-screen intervals, blinded outcome adjudication, reliability testing, and prospective evaluation of workflow and cost.

This observational, single-system study is susceptible to residual confounding and spectrum bias despite statistical adjustment, and generalizability may be constrained by local case-mix and infection surveillance practices. Screening timing and re-screen cadence were not fully standardized, introducing potential misclassification. Interrater reliability for STRONGkids and PYMS was not prospectively quantified. Missing data and reliance on parent-reported weight or intake may have introduced measurement error, and ascertainment of hospital-acquired infection using routine surveillance may have underdetected events. Several subgroup comparisons, including infants and digestive disease, were underpowered. The proposed decision thresholds and pathway were not prospectively tested, and process metrics such as time-to-complete, staffing burden, workflow disruption, and costs were not measured. Use of WHO criteria as the reference standard may affect calibration relative to alternative definitions, and lack of blinding to screening scores may have introduced observer or incorporation bias.

## 6 Conclusion

The modified STRONGkids tool demonstrated superior discrimination for WHO-defined malnutrition compared with PYMS and stratified downstream risk for hospital-acquired infection and length of stay. Risk gradients were most pronounced among infants younger than 1 year and children with digestive diseases. We operationalize these findings into a pragmatic screening-to-action pathway: STRONGkids scores of 4–5 prompt same-day dietitian assessment, baseline laboratory evaluation, initiation of nutrition support, and re-screening at 48–72 h; scores of 1–3 accompanied by digestive disease or age younger than 1 year are managed as functionally high risk. These steps are feasible on general pediatric wards and provide immediate guidance for targeted escalation and scheduled reassessment. External validation and prospective impact evaluation are warranted.

## Data Availability

The original contributions presented in the study are included in the article/Supplementary Material, further inquiries can be directed to the corresponding author.
